# Mapping network connection and direction among symptoms of depression and anxiety in patients with chronic gastritis

**DOI:** 10.1002/pchj.757

**Published:** 2024-04-14

**Authors:** Qihui Tang, Rui Wang, Haiqun Niu, Yifang Li, Yuting Li, Zichao Hu, Xiangping Liu, Yanqiang Tao

**Affiliations:** ^1^ Faculty of Psychology Beijing Normal University Beijing China; ^2^ Beijing Key Laboratory of Applied Experimental Psychology, National Demonstration Center for Experimental Psychology Education Beijing Normal University Beijing China; ^3^ Department of Gastroenterology The First Affiliated Hospital of Anhui University of Chinese Medicine Hefei China; ^4^ School of Psychology Nanjing Normal University Nanjing China; ^5^ Department of Chinese Medicine Nursing, School of Nursing Anhui University of Chinese Medicine Hefei China; ^6^ School of Psychology Shanghai University of Sport Shanghai China

**Keywords:** anxiety, chronic gastritis, depression, network analysis

## Abstract

Regarding neurophysiological and developmental findings, anxiety and depression are usual comorbidities of gastritis patients. However, research related to anxiety and depression among chronic gastritis patients was conducted on the disease level while ignoring symptoms. Hence, we rendered the network approach to reveal the symptoms of anxiety and depression among chronic gastritis patients. Three hundred and sixty‐nine chronic gastritis patients (female = 139, *M*_*age*_ *=* 55.87 years) were asked to complete the Self‐Rating Anxiety Scale and Self‐Rating Depression Scale. Three symptom networks and one directed acyclic graph (DAG) network were formed. First, in the anxiety network of chronic gastritis patients, *dizziness* was the most influential symptom. In the depression network of chronic gastritis patients, *depressed affect* and *psychomotor retardation* were the influential symptoms. Second, *panic, easy fatiguability, weakness, palpitation, depressed affect, tachycardia, fatigue*, and *psychomotor agitation* bridged the anxiety–depression network of chronic gastritis patients. Third, DAG networks showed that *anxiousness* and *hopelessness* could trigger other symptoms in the anxiety–depression networks of chronic gastritis patients. The current study provided insightful information on patients with chronic gastritis by examining the structures of symptoms.

## INTRODUCTION

Chronic gastritis refers to a common condition characterized by persistent, but low‐grade, inflammation of the stomach mucosa, which is typically divided into atrophic and non‐atrophic gastritis (Dixon et al., 1996; Polydorides, 2014). As one of the most common life‐long and insidious illnesses, chronic gastritis is worth our vigilance since it is often recognized as a precursor to gastric cancer, which is the fifth most common cancer and the third leading cause of cancer mortality worldwide (Yang et al., 2022). Most cases of chronic gastritis are caused by *Helicobacter pylori* infection, and other causes include an unhealthy diet, imbalanced intestinal flora, abnormalities in the microbiota–gut–brain axis, and familial pathological background, among others (Banks et al., 2019; Margolis et al., 2021; Sukocheva et al., 2020). Recently, a meta‐analysis indicated that the prevalence of chronic atrophic gastritis worldwide was about 25% (Yin et al., 2022). Moreover, the prevalence of chronic gastritis varies by country and region. For instance, chronic atrophic gastritis affects approximately 15% of the U.S. population (Shah et al., 2021), while in China, 24.48% of patients undergoing gastroscopy screening are diagnosed with chronic gastritis (Gong et al., 2022). However, there are even estimates in the literature that perhaps more than half of the world's population suffers from this disease to some degree (Sipponen & Maaroos, 2015).

It is worth noting that several studies have shown that individuals with chronic gastritis have a high prevalence of depression and anxiety (Özyurt et al., 2019; Wu et al., 2022). Specifically, depression is a complex mental health disorder made up of a range of symptoms, such as low mood, guilt, and worthlessness (American Psychiatric Association, 2013). Anxiety, a common comorbid mental condition of depression, refers to the anticipation of future concerns and is associated with symptoms such as excessive worrying and nervousness (Beard et al., 2016). It has been proven that patients with chronic gastritis have a higher rate of anxiety disorders (27.0% vs. 15.3%) and affective disorders (20.1% vs. 11.5%) compared to non‐patients (Goodwin et al., 2013). This high inclination may be related to long‐term treatment, as studies have shown that patients with chronic gastritis have to suffer long‐term treatment due to the recurrence of the disease, which in turn leads to anxiety and depression (Özyurt et al., 2019; Wu et al., 2022). These pathological problems in individuals with chronic gastritis require great attention and urgent intervention because mental health problems affect not only well‐being (Lin et al., 2021) but also treatment outcomes (Borsboom & Cramer, 2013).

The biopsychosocial model offers the latest and most comprehensive framework for understanding and treating patients with mental health problems. This model, first introduced by Engel (1977, 1980), highlights the need for modern medicine to adopt a more dynamic systems approach that includes different factors from sociocultural and psychological levels, not just those factors related to patients and physicians. In other words, this model emphasizes that disease is not exclusively a disorder that occurs at the biological level, but that psychological and social effects are equally important (Gatchel et al., 2007; Havelka et al., 2009). Accordingly, treating patients with gastritis requires not only the treatment of the gastritis symptoms but also attention to their mental health problems. Inspired by the biopsychosocial model, scholars have begun to explore the psychiatric features among patients affected by chronic gastrointestinal diseases and clarify the connection between psychopathology disorders and such diseases (Addolorato et al., 2008).

Additionally, psychopathology disorders and chronic gastritis may have a bidirectional relationship, which indicates the existence of a negative cycle between chronic gastritis and psychopathology disorders. Specifically, malignant changes in the gut–brain axis, which maintain homeostasis of bodies, have been proven to be the root of mental disorders and chronic gastritis, indicating that alterations of brain signals can induce unhealthy diets and gut inflammation (Arneth, 2018). Conversely, such perturbations of gut signals may alter the activations of brain regions and impair cognitive functions (Mayer, 2011). More recently, Sharon et al. (2016) found that disruption of the gut microbiome contributes to mental disorders, including autism spectrum disorder in children, schizophrenia, anxiety, and depression. With the recognition of the importance of the microbiome, the microbiome–gut–brain axis was established to further explain the bidirectional relationship between chronic gastritis and the comorbidities of anxiety and depression (Wang & Kasper, 2014). To sum up, people with chronic gastritis are more likely to develop anxiety and depression disorders, and this, in turn, will aggravate their chronic gastritis, forming a negative cycle that needs to be broken.

Given that people suffering from chronic gastritis are accompanied by a bad mental state of anxiety and depression, several depression rehabilitation methods have been suggested to relieve gastritis patients' suffering (Dash et al., 2015). Specifically, mindfulness (van Agteren et al., 2021) and moving to emptiness technique (Tao et al., 2022) are recommended to alleviate pain in the gut and psychological abnormalities. However, although several methods are used to ease the symptoms of chronic gastritis, studies on the mental health of patients with chronic gastritis have ignored the fact that symptoms play a dynamic role in anxiety and depression manifestation (Marchetti, 2019). This statement comes from the emerging network theory of mental disorders, which proposes that mental disorders are multi‐dimensional and consist of different dynamic symptoms (Borsboom, 2017). According to this theory, symptoms constitute mental disorders, rather than being reflective of the disorders, and these symptoms can cause each other in feedback loops that settle into self‐sustaining equilibria (Borsboom & Cramer, 2013). In this view, to treat or prevent a disorder, a more effective way is to therapeutically target certain symptoms that can cause other symptoms (Jones et al., 2018). For instance, chronic gastritis patients with an anxiety disorder may be prescribed medicine to treat all visible symptoms, such as nausea, pain, worry, and fear (Borsboom & Cramer, 2013). However, the truth is that the patient may be mainly beset with uncontrollable worry about body changes and frequently changing diets. When the critical symptom of “uncontrollable worry about body figure” is alleviated, the patient is likely to obtain more efficient treatment and recovery. However, most previous studies only analyze mental disorders as a single dimension and cover up the complex relationship between symptoms and efficient intervention on symptoms. Therefore, it is necessary to examine specific anxiety and depression symptoms and their interactions among patients with a newly proposed method, that is, the network analysis.

The network analysis helps researchers investigate the most critical symptoms (nodes) that can connect or stimulate several symptoms of mental disorders and the most interconnected symptoms (edges), which indicate a causal relationship (i.e., partial correlation or regression coefficients) or a common etiological influence (Borsboom, 2017; Tao, Niu, et al., 2023; Tao, Tang, et al., 2023; Triolo et al., 2021). In this way, it will help to find efficient interventions for anxiety and depression disorders, thereby benefiting the treatment of chronic gastritis. To the best of our knowledge, no network analysis has been performed to reveal typical symptoms of anxiety and depression among chronic gastritis patients. Hence, in the current study, anxiety and depression symptom structures for chronic gastritis patients were formed to identify: (i) critical connections of pairwise symptoms that mutually strengthen; (ii) core symptoms in anxiety and depression networks, respectively; (iii) bridge symptoms that could link anxiety and depression; and (iv) triggered symptoms in the anxiety–depression network.

## METHOD

### Participants

This cross‐sectional survey was conducted in March 2022. All participants were diagnosed with chronic gastritis at the First Affiliated Hospital of Anhui University of Chinese Medicine.

The inclusion criteria were as follows: (1) met diagnostic criteria for chronic gastritis based on the Chinese Consensus on Chronic Gastritis in Shanghai, China (Fang et al., 2018); (2) were over 18 years old, without gender restrictions; and (3) attended the test with informed consent.

Patients were excluded if they: (1) had physical conditions that fluctuated for a peptic ulcer, a tumor, a pyloric obstruction, or gastrointestinal hemorrhage; (2) had health conditions that may diminish a combination of heart, lung, brain, kidney, and other critical disorders; (3) had other ailments; (4) used anti‐anxiety or anti‐depression medications; (5) were pregnant or weaning; (6) refused to or could not complete the questionnaire; (7) failed to provide completely informed consent owing to cognitive or behavioral disability; (8) had poor medication adherence, short‐term discharges, or frequent travel; (9) had severe skin lesions or medication allergies; or (10) were in clinical trials of other drugs.

Given the current recruitment protocol employed by physicians during outpatient visits and considering the aforementioned inclusion and exclusion criteria, as well as the personal preferences of participants, only individuals who met the criteria for inclusion in the present study were enrolled. Consequently, the study consisted of a sample size of 369 patients diagnosed with chronic gastritis, including 139 (37.67%) females. The average age was 55.87 years (*SD* = 12.37 years, range: 28–82 years). The majority of participants were married (*n* = 350, 94.85%), and approximately half resided in urban areas (*n* = 208, 56.37%). Detailed sociodemographic characteristics of all participants are presented in Table 1.

**TABLE 1:** Sociodemographic characteristics of the patients with chronic gastritis.

Variable	Chronic gastritis (*N* = 369)
Gender	
Male	230 (62.33%)
Female	139 (37.67%)
Marriage	
Divorced	3 (0.81%)
Married	350 (94.85%)
Single	4 (1.08%)
Widowed	12 (3.25%)
Mean age (years)	55.87 (*SD* = 12.37)
Residence	
City	208 (56.37%)
Rural	161 (43.63%)

The ethics committee of Anhui University of Chinese Medicine approved this study protocol (Reference number: 2022MCZQ04). Patients who signed the written informed consent were asked to complete and return a battery of self‐report scales as well as provide demographic information, including gender, marital status, age, and residence, to the researchers immediately. Clinicians would intervene based on the patient's score.

### Measures

#### Self‐rating Anxiety Scale

The Self‐rating Anxiety Scale (SAS) is widely used to measure anxiety levels in adults and adolescents (Zung, 1971) with 20 self‐report items (4‐point Likert‐type scale). Higher values on the scale indicate a greater inclination toward anxiety. The Chinese version has been proven to be valid (Ning et al., 2020). The Cronbach's *α* value for the SAS of the current study was .78.

#### Self‐rating Depression Scale

Zung (1965) designed the Self‐rating Depression Scale (SDS) to measure depressive levels with 20 self‐report items (4‐point Likert‐type scale). A higher mean value on the scale indicates a depressive inclination. The Chinese version has been validated (Liu et al., 2020). The Cronbach's α value for the SDS of the present study was .82.

### Statistical procedure

All analyses were performed in R software. We used the *formattable* package (Ren & Russell, 2016) to calculate the mean, standard deviation (*SD*), skewness, and kurtosis of all SAS and SDS items.

#### Network estimation

In network analysis, each symptom (i.e., questionnaire item) was depicted as a “node,” while the relationships between symptoms were termed “edges.” The thickness of edges, as per graph theory (Epskamp et al., 2012), reflects the strength of connections between nodes, with green denoting positive correlations and red indicating negative correlations. To construct the network models and evaluate their structures, we utilized the *bootnet* package (Epskamp et al., 2018). Specifically, we employed the extended Bayesian Information Criterion (EBIC; Chen & Chen, 2008) graphical least absolute shrinkage and selection operator (LASSO) network models to calculate the network structure of depression–anxiety symptoms (Epskamp & Fried, 2018). This approach facilitated the creation of a sparse network model, effectively minimizing spurious correlations by reducing certain coefficients to zero.

Strength, representing the absolute sum of edge weights connected to a node, served as a centrality index to assess the significance of symptoms within the anxiety or depression network structure (Bringmann et al., 2019). Similarly, bridge strength, defined as the sum of the absolute value of all edges between node A and all nodes not in the same community as node A, was computed to identify bridge symptoms linking anxiety and depression (Jones et al., 2018). The R packages “*qgraph*” (Epskamp et al., 2012) and “*networktools*” (Jones, 2018) were utilized to calculate these indices.

#### Estimation of network stability and accuracy

Four procedures were employed to assess the stability and accuracy of the network structure (Epskamp et al., 2018). First, the accuracy of edge weights was evaluated using confidence intervals (CIs) obtained through the nonparametric bootstrapping method. The data were randomly resampled to generate new datasets from which 95% of CIs were derived. Larger CIs indicated a less precise estimation of the edges, while smaller CIs indicated greater precision (Epskamp et al., 2018). Second, in subset bootstraps, the correlation stability coefficient (CS‐C) was applied to measure the stability of centrality indices (i.e., strength and bridge strength; Jones, 2018; Valente, 2012). The CS‐C represented the maximum proportion of samples that could be removed, with a 95% probability that the correlation between the original centrality indices would be at least 0.70 (Epskamp et al., 2018). Moreover, a CS‐C value should be greater than 0.25 and preferably greater than 0.5 (Epskamp et al., 2018). Third, bootstrapped difference tests were employed to analyze differences in network properties (Epskamp et al., 2018). The significance of the difference between strength and edges was determined if the nonparametric 95% CIs for 1000 bootstrap samples did not encompass zero (Costenbader & Valente, 2003). Finally, predictability was assessed using the R package “*mgm*” (Haslbeck & Waldorp, 2020) to evaluate how effectively neighbors of all nodes predict a single node.

#### Directed Acyclic Graph analysis

In contrast to EBIC networks, which are based on partial correlations, a Directed Acyclic Graph (DAG) is a Bayesian network with directed edges and without cycles. A DAG reveals trigger symptoms that can cause other symptoms and the degree to which strong trigger symptoms can activate other symptoms (Jones et al., 2018). The current study used *bnlearn*, the R package, to construct DAGs for chronic gastritis patients (McNally, 2016). The completed partial DAG can make up for some shortcomings in equivalent separate DAGs (Scutari Marco, 2021; Tao, Hou, et al., 2023).

## RESULTS

### Item description

The mean, *SD*, skewness, and kurtosis of anxiety and depressive symptoms measured by the SAS and SDS are shown in Tables S1 and S2 for chronic gastritis patients.

### Network structure and centrality index

**FIGURE 1:**
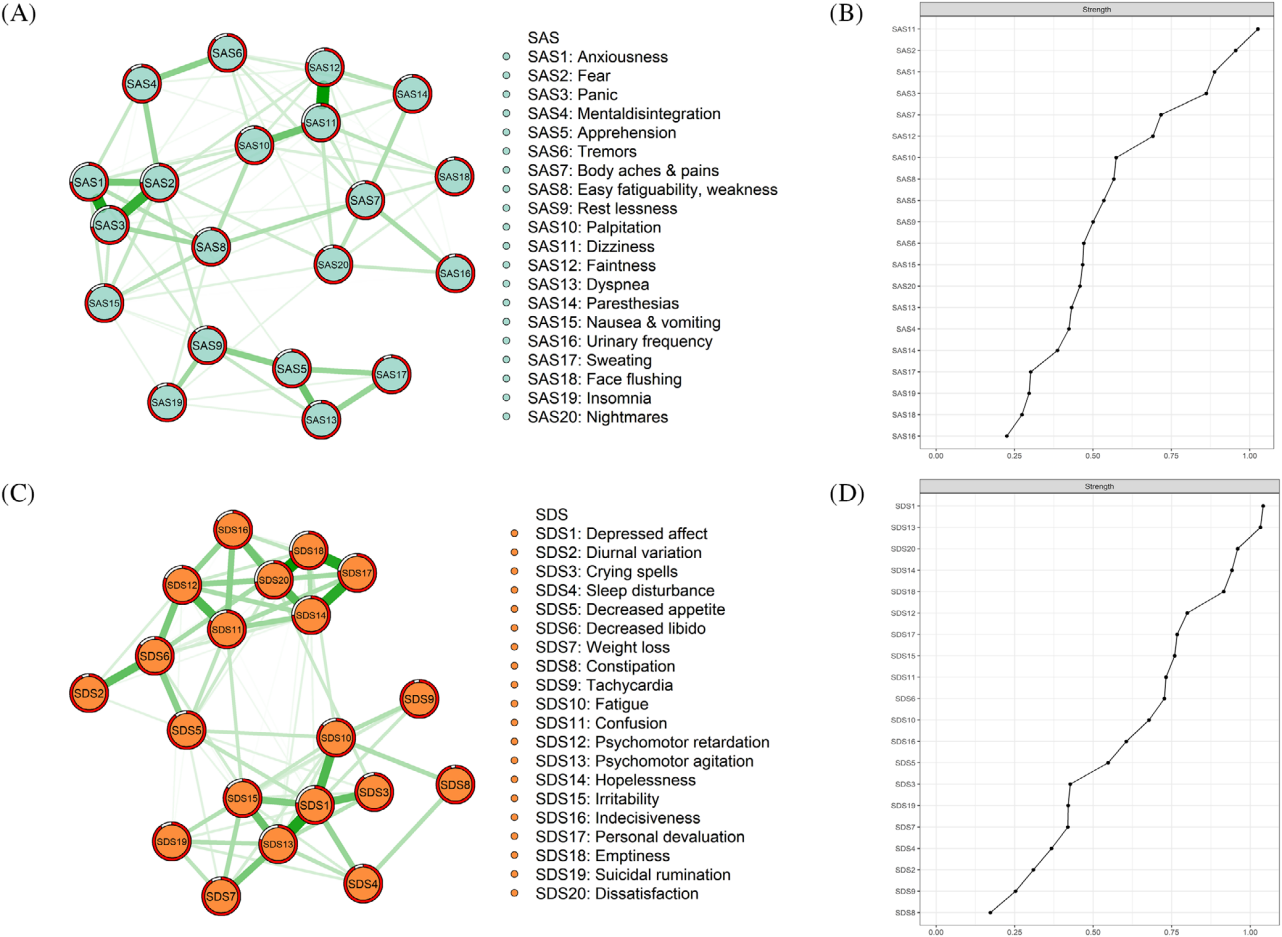
The network structure and standardized centrality indices of anxiety and depressive symptoms among chronic gastritis patients. (A) Anxiety network of chronic gastritis patients. (B) Centrality index of anxiety among chronic gastritis patients. (C) Depression network of chronic gastritis patients. (D) Centrality index of depression among chronic gastritis patients.

The network structures of anxiety and depression among chronic gastritis patients are shown in Figure 1A,C. First, 75 edges (39.47%) and 84 edges (44.21%) were not zero among 190 possible edges for anxiety and depressive networks, respectively (see Tables S2 and S3 in the supplement). Furthermore, the two strongest edges for anxiety were SAS11 (*dizziness*)—SAS12 (*faintness*) and SAS2 (*fear*)—SAS3 (*panic*). In the depressive network, SDS1 (*depressed affect*)—SDS13 (*psychomotor agitation*) and SDS18 (*emptiness*)—SDS20 (*dissatisfaction*) were the two strongest edges.

**FIGURE 2:**
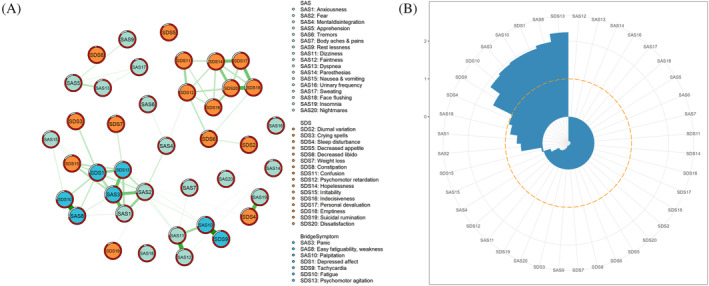
The bridge network of anxiety–depression symptoms among chronic gastritis patients. (A) Bridge network of chronic gastritis patients. (B) Bridge centrality index of anxiety–depression among chronic gastritis patients.

Strength is an essential index in network analysis, and several points were mentioned. As shown in Figure 2B,D, SAS11 (*dizziness*) was the node with the most centrality (*Z* ≥ 1) in the anxiety network. Moreover, nodes (*Z* ≥ 1), SDS1 (*depressed affect*), and SDS13 (*psychomotor agitation*) had the greatest centrality in the depression network.

### Bridge network structure and bridge centrality

In addition to two single anxiety and depression networks for chronic gastritis patients, we constructed the comorbidity network, including both anxiety and depression. The index of the bridge's strength was used to indicate the co‐occurring mechanisms of anxiety and depression.

In the comorbidity network of chronic gastritis patients, SAS3 (*panic*), SAS8 (*easy fatiguability, weakness*), SAS10 (*palpitation*), SDS1 (*depressed affect*), SDS9 (*tachycardia*), SDS10 (*fatigue*), and SDS13 (*psychomotor agitation*) had the standardized bridge strength values > 1, as shown in Figure 2.

### Network stability and accuracy

**FIGURE 3:**
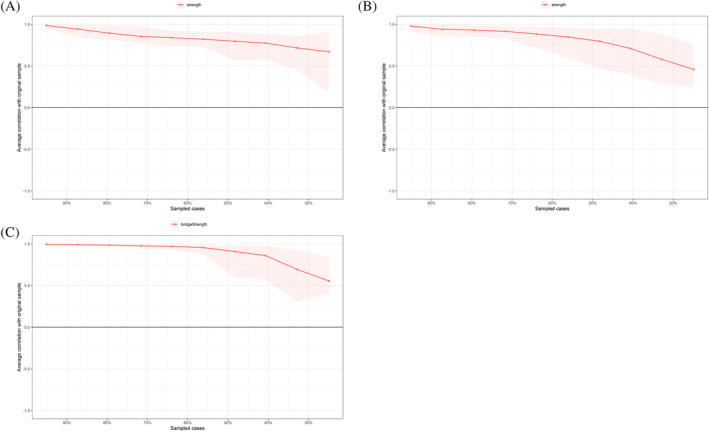
The *x*‐axis indicates the percentage of cases of the original sample included at each step. The *y*‐axis indicates the average of correlations between the centrality indices from the original network and the centrality indices from the networks that were re‐estimated after excluding increasing percentages of cases. (A) Anxiety for patients with chronic gastritis. (B) Depression for patients with chronic gastritis. (C) Bridge network of chronic gastritis patients.

The bootstrapped CIs and bootstrapped difference tests for the edge weights are shown in Figures S1 and S2, indicating that all current networks were stable. Figure S3 indicates the high stability of the centrality estimates. The case‐dropping subset bootstrap procedure showed that the strength indices remained stable after dropping large sample proportions (see Figure 3). Moreover, strength reported high stability (i.e., *CS‐C* = 0.43; *CS‐C* = 0.36; *CS‐C* = 0.44) for the anxiety, depression, and bridge network of chronic gastritis patients.

The predictability index was used to indicate the network's accuracy. Predictability refers to how well a given node can be predicted by its neighboring nodes. On average, in single anxiety and depressive networks of chronic gastritis patients, 86% and 85% of the variance (predictability) could be accounted for by each node's neighbors (*M*_predictability_ = 0.86 ± 0.07; *M*_predictability_ = 0.85 ± 0.07). Similarly, for the bridge network of chronic gastritis patients, the predictabilities were 0.82 (*M*_*predictability*_ = 0.82 ± 0.08).

### DAG analysis

**FIGURE 4:**
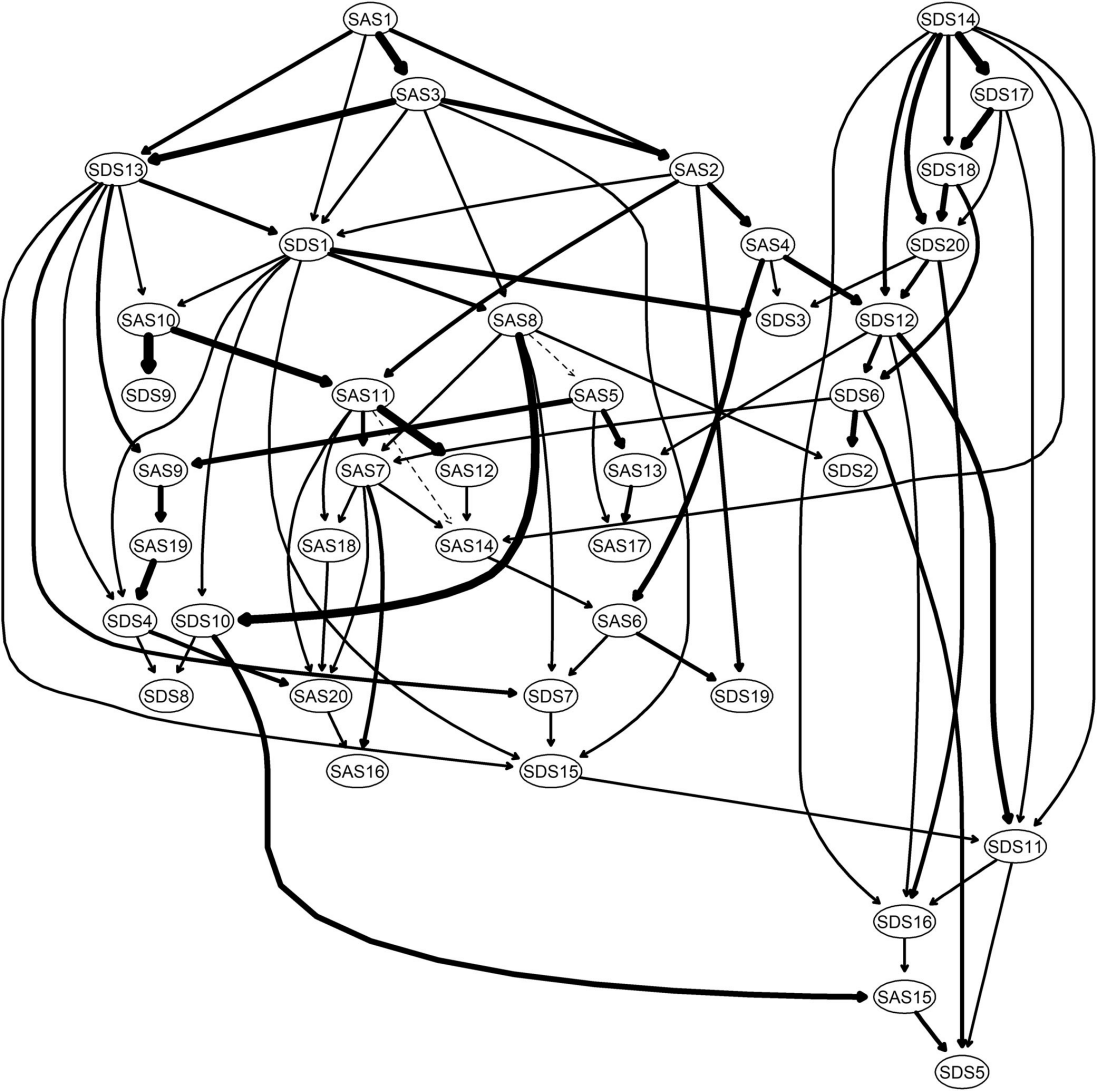
Anxiety–depression Directed Acyclic Graph of chronic gastritis patients. Nodes represent symptoms, and edges represent pairwise symptoms.

In Figure 4, SDS14 (*hopelessness*) and SAS1 (*anxiousness*) trigger symptoms in chronic gastritis patients' DAG networks. Anxiety and depression symptoms in the DAG network are mainly crosswise, implying that anxiety and depression symptoms among chronic gastritis patients are complex. However, we can find that *hopelessness* and *anxiousness* triggered seven and four other symptoms without being triggered by any other higher‐level symptoms, respectively. *Hopelessness* strongly motivated SDS17 (*personal devaluation*), and *anxiousness* strongly activated SAS3 (*panic*). Meanwhile, SDS5 (*decreased appetite*) was the most downstream symptom in the DAG network.

## DISCUSSION

We obtained data from 369 chronic gastritis patients and described the anxiety and depression networks, respectively, in detail. The core symptoms in the single network of anxiety and depression, the bridge symptoms that link anxiety and depression, and the triggered symptoms in the anxiety–depression network were also confirmed. Some results are discussed below.

For chronic gastritis patients, the most conspicuous anxiety symptom was *dizziness*, a frequent symptom of anxiety that can increase treatment frequency (Wiltink et al., 2009). This is not quite in line with previous research on general populations, showing that *panic*, *anxiousness*, and *fear* are quotidian symptoms (Addolorato et al., 2008; Clayton et al., 2013; Craske et al., 2017; Dymond et al., 2015). It also differs from the results of another study, which found that patients with chronic pain demonstrated excessive fear (Meulders, 2020). Chronic gastritis is a long‐term disease that can evolve into dyspepsia and even gastritis cancer, which undoubtedly leads to more severe anxiety (Macke et al., 2022). This is coupled with the fact that patients with chronic gastritis tend to acquire the pattern of catastrophizing symptoms of nausea or vomiting, which can easily trigger feelings of dizziness (Carleton, 2012). Therefore, it is unsurprising that dizziness is the most central symptom of anxiety among people with chronic gastriti*s*.

In the depressive symptoms of patients with chronic gastritis, *depressed affect* was found to be the most central symptom, followed by *psychomotor retardation*. These findings are consistent with their current status as hallmark symptoms required for a diagnosis of depression and with prior studies (Fried & Nesse, 2014; Fried & Nesse, 2015). The fact that the symptom of *depressed affect* is the most central to the network is similar to Beard et al.'s 2016 finding that *sad mood* is the most central to the network of depressive symptoms. However, Beard et al.'s study on the network of depression and anxiety symptoms among psychiatric patients used the Patient Health Questionnaire (PHQ‐9), while our study investigated depressive symptoms in chronic gastritis patients using the SDS. Despite different study measures and samples, our findings that depressive manifestations in patients with chronic gastritis can be narrowed down to the most central emotional issue provide clues for relevant intervention, diagnosis, and treatment. The second central symptom in the depression network, *psychomotor retardation*, a slowing of mental and motor activity, has been proven to be a hallmark neuro‐vegetative symptom of depression that can be explained by stress and inflammation (Slavich & Irwin, 2014). Given that aging itself already causes substantial psychomotor slowing in healthy elderly individuals (Seidler et al., 2010), these elderly individuals suffering from chronic gastritis can easily feel exhausted by the recurrent attacks of this disease that bring about nausea or weakness.

As for the symptoms that bridge the anxiety–depression network of chronic gastritis patients, seven different symptoms—panic, easy fatiguability, weakness, palpitation, depressed affect, tachycardia, fatigue, and psychomotor agitation — have shown high bridge values. That is, patients who feel panicky, fatigued, weak, or palpitating about their anxiety would be at a greater risk for depression. Take *panic* as an example. *Panic*, as a quotidian symptom of anxiety, is mainly induced by intolerance of uncertainties and high anxiety sensitivity (Carleton et al., 2013). Patients with chronic gastritis, a long‐lasting disease that may evolve into gastritis cancer (Macke et al., 2022), will undoubtedly be frightened about their health and lives. Such a state can obviously elicit depressive feelings in patients and then may develop into depression. In summary, interventions targeting bridge symptoms might be more efficient and precise than overall psychotherapeutic interventions in altering the interconnectedness between the subnetworks of depression and anxiety (Kaiser et al., 2021).

Another finding that must be highlighted is that *anxiousness* and *hopelessness* could trigger other symptoms in anxiety–depression networks of chronic gastritis patients. *Anxiousness*, a hackneyed symptom of anxiety, is an endurable trait that could induce isolation, avoidance, and loneliness (Addolorato et al., 2008; Clayton et al., 2013). Hopelessness refers to the belief that nothing can be done to change the pessimistic scenario when one finds that negative events will occur and/or positive events will not occur (Abramson et al., 1989). Our findings are partially consistent with the results of a previous study, which found that high levels of hopelessness were associated with depression (Marchetti et al., 2016). As a long‐lasting chronic and active inflammation, chronic gastritis cannot be harmless and will result in damage to gastric function and even gastric cancer (Sipponen & Maaroos, 2015). This means that those with chronic gastritis need to worry about the possibility of getting gastric cancer while receiving long‐term treatment. Furthermore, such a disease is not only related to the body, but also means a considerable financial burden on the family. Therefore, we speculate that the sense of anxiety among these elderly people is not only due to the disease itself, but also to financial reasons (Huang et al., 2024). As for their hopelessness, it may be more related to the fact that although their physiological functions are getting weaker day by day, they still have to suffer from this disease for the rest of their lives. In this way, it is reasonable that the anxiety–depression network can be triggered by *anxiousness* and *hopelessness*. Taken together, findings from our study further confirmed that patients with chronic gastritis were at high risk of psychological distress (Jiang et al., 2009).

## LIMITATIONS

This study featured several limitations. First, a cross‐sectional study cannot explore or reveal the dynamic process and causal relationship among symptoms of anxiety or depression. In the future, longitudinal data should be collected and analyzed. Second, we collected data from 369 patients. To conduct a more insightful study, more participants should be examined. Third, the meticulous classification of different periods of gastritis should be considered to reveal the evolution of anxiety and depression in different disease courses (Sibelli et al., 2016; Thabane et al., 2007). Fourth, although age was regarded as a covariate in our study, a cohort study should be conducted to avoid confounding effects (Pilotto & Malfertheiner, 2002).

## CONCLUSION

In summary, this study is the first to analyze and compare the anxiety and depression symptom networks among chronic gastritis patients. We found that *dizziness* in the anxiety network, and *depressed affect* and *psychomotor retardation* in the depression network were the most central symptoms. Although we identified eight symptoms bridging the anxiety–depression network of chronic gastritis patients, *anxiousness* and *hopelessness* showed their central effect triggering other symptoms in the anxiety–depression network. The results may shed some light on exploring the impact of chronic gastritis on anxiety and depression. The central and bridge symptoms identified may be useful for depression and anxiety treatment. In addition, findings of chronic gastritis patients' anxiety and depression symptoms can guide the treatment of their mental health symptoms.

## CONFLICT OF INTEREST STATEMENT

The authors declare there are no conflicts of interest.

## ETHICS STATEMENT

The research was examined and approved by the ethics committee of Anhui University of Chinese Medicine (Reference number: 2022MCZQ04).

## INFORMED CONSENT

Informed consent was obtained from all individual participants included in the study.

## Supporting information


**Table S1.** Means, standard deviations, skewness, and kurtosis for variables among patients with chronic gastritis (*N* = 369).
**Table S2.** Weighted adjacency matrix for anxiety symptoms among patients with chronic gastritis.
**Table S3.** Weighted adjacency matrix for depression symptoms among patients with chronic gastritis.
**Figure S1.** Nonparametric bootstrapped confidence intervals of estimated edges. The red line represents the estimated edge, while the shaded area indicates the 95% bootstrap confidence interval. A, anxiety for patients with chronic gastritis; B, depression for patients with chronic gastritis; C, anxiety–depression for chronic gastritis groups.
**Figure. S2.** Nonparametric bootstrapped difference test for edges. Grey boxes indicate no significant difference, whereas black boxes indicate a statistically significant difference (*p* < .05). Diagonal color and saturation represent the magnitude and direction of each estimated edge. A, anxiety for patients with chronic gastritis; B, depression for patients with chronic gastritis; C, anxiety–depression for chronic gastritis groups.
**Figure. S3.** The stability difference tests (α = .05) for “node strength.” Grey boxes reflect no significant differences, and black boxes reflect significant differences. The number in the white boxes (i.e., the diagonal line) denotes the value of the node strength of a specific node. A, anxiety for patients with chronic gastritis; B, depression for patients with chronic gastritis; C, anxiety–depression for chronic gastritis groups.

## Data Availability

Analytic code and data for this work are available upon request.
