# Supplementary material for: Mapping network connection and direction among symptoms of depression and anxiety in patients with chronic gastritis
Source: Psych J. 2024 Apr 14;13(5):824–34. doi: 10.1002/pchj.757 (PMC11444727; doi:10.1002/pchj.757)
Supplement: Supplementary file 1 — Table S1. Means, standard deviations, skewness, and kurtosis for variables among patients with chronic gastritis (N = 369). Table S2. Weighted adjacency matrix for anxiety symptoms among patients with chronic gastritis. Table S3. Weighted adjacency matrix for depression symptoms among patients with chronic gastritis. Figure S1. Nonparametric bootstrapped confidence intervals of estimated edges. The red line represents the estimated edge, while the shaded area indicates the 95% bootstrap confidence interval. A, anxiety for patients with chronic gastritis; B, depression for patients with chronic gastritis; C, anxiety–depression for chronic gastritis groups. Figure. S2. Nonparametric bootstrapped difference test for edges. Grey boxes indicate no significant difference, whereas black boxes indicate a statistically significant difference (p < .05). Diagonal color and saturation represent the magnitude and direction of each estimated edge. A, anxiety for patients with chronic gastritis; B, depression for patients with chronic gastritis; C, anxiety–depression for chronic gastritis groups. Figure. S3. The stability difference tests (α = .05) for “node strength.” Grey boxes reflect no significant differences, and black boxes reflect significant differences. The number in the white boxes (i.e., the diagonal line) denotes the value of the node strength of a specific node. A, anxiety for patients with chronic gastritis; B, depression for patients with chronic gastritis; C, anxiety–depression for chronic gastritis groups. [file PCHJ-13-824-s001.docx]

Table S1. Means, standard deviations, skewness, and kurtosis for variables among patients with chronic gastritis (*N* = 369).

| Variable | *Mean* | *SD* | *Skew* | *Kurtosis* |
| --- | --- | --- | --- | --- |
| SAS1 | 1.93 | 0.98 | 0.72 | -0.56 |
| SAS2 | 1.77 | 0.87 | 0.88 | -0.13 |
| SAS3 | 1.83 | 0.88 | 0.79 | -0.26 |
| SAS4 | 1.29 | 0.66 | 2.41 | 5.26 |
| SAS5 | 2.73 | 1.13 | -0.31 | -1.31 |
| SAS6 | 1.26 | 0.63 | 2.67 | 4.97 |
| SAS7 | 1.85 | 0.98 | 0.89 | -0.33 |
| SAS8 | 2.17 | 1.02 | 0.46 | -0.91 |
| SAS9 | 2.32 | 1.13 | 0.26 | -1.33 |
| SAS10 | 1.60 | 0.76 | 1.23 | 1.17 |
| SAS11 | 1.60 | 0.83 | 1.38 | 1.26 |
| SAS12 | 1.36 | 0.70 | 2.22 | 4.70 |
| SAS13 | 2.19 | 1.30 | 0.41 | -1.59 |
| SAS14 | 1.47 | 0.77 | 1.75 | 2.58 |
| SAS15 | 2.57 | 1.09 | -0.05 | -1.30 |
| SAS16 | 1.89 | 1.02 | 0.83 | -0.57 |
| SAS17 | 2.55 | 1.22 | -0.03 | -1.58 |
| SAS18 | 1.32 | 0.70 | 2.43 | 4.59 |
| SAS19 | 2.67 | 1.18 | -0.19 | -1.49 |
| SAS20 | 1.75 | 0.96 | 1.11 | 0.14 |
| SDS1 | 1.85 | 0.95 | 0.78 | -0.54 |
| SDS2 | 2.39 | 1.12 | 0.20 | -1.32 |
| SDS3 | 1.32 | 0.67 | 2.09 | 3.62 |
| SDS4 | 2.33 | 1.13 | 0.19 | -1.37 |
| SDS5 | 2.40 | 1.18 | 0.13 | -1.48 |
| SDS6 | 2.46 | 1.21 | 0.03 | -1.57 |
| SDS7 | 1.93 | 1.15 | 0.80 | -0.90 |
| SDS8 | 1.70 | 1.00 | 1.13 | -0.10 |
| SDS9 | 1.46 | 0.75 | 1.61 | 1.92 |
| SDS10 | 2.10 | 1.01 | 0.49 | -0.91 |
| SDS11 | 1.98 | 1.06 | 0.60 | -1.01 |
| SDS12 | 2.21 | 1.16 | 0.32 | -1.39 |
| SDS13 | 1.75 | 0.95 | 1.02 | -0.10 |
| SDS14 | 1.97 | 1.05 | 0.65 | -0.89 |
| SDS15 | 2.02 | 0.98 | 0.54 | -0.81 |
| SDS16 | 2.42 | 1.08 | 0.07 | -1.27 |
| SDS17 | 2.00 | 1.08 | 0.68 | -0.88 |
| SDS18 | 2.17 | 1.07 | 0.35 | -1.20 |
| SDS19 | 1.31 | 0.71 | 2.37 | 4.95 |
| SDS20 | 2.05 | 1.08 | 0.57 | -1.01 |

Table S2. Weighted adjacency matrix for anxiety symptoms among patients with chronic gastritis.

|  | SAS1 | SAS2 | SAS3 | SAS4 | SAS5 | SAS6 | SAS7 | SAS8 | SAS9 | SAS10 | SAS11 | SAS12 | SAS13 | SAS14 | SAS15 | SAS16 | SAS17 | SAS18 | SAS19 | SAS20 |
| --- | --- | --- | --- | --- | --- | --- | --- | --- | --- | --- | --- | --- | --- | --- | --- | --- | --- | --- | --- | --- |
| SAS1 | 0.000 | 0.182 | 0.301 | 0.075 | 0.000 | 0.000 | 0.000 | 0.097 | 0.055 | 0.062 | 0.029 | 0.013 | 0.000 | 0.000 | 0.074 | 0.000 | 0.000 | 0.000 | 0.000 | 0.000 |
| SAS2 | 0.182 | 0.000 | 0.267 | 0.134 | 0.033 | 0.000 | 0.000 | 0.000 | 0.074 | 0.000 | 0.069 | 0.059 | 0.000 | 0.000 | 0.076 | 0.000 | 0.000 | 0.000 | 0.000 | 0.061 |
| SAS3 | 0.301 | 0.267 | 0.000 | 0.012 | 0.000 | 0.024 | 0.022 | 0.121 | 0.000 | 0.011 | 0.039 | 0.000 | 0.000 | 0.000 | 0.064 | 0.000 | 0.000 | 0.000 | 0.000 | 0.000 |
| SAS4 | 0.075 | 0.134 | 0.012 | 0.000 | 0.000 | 0.151 | 0.000 | 0.000 | 0.000 | 0.000 | 0.000 | 0.030 | 0.000 | 0.000 | 0.000 | 0.000 | 0.000 | 0.022 | 0.000 | 0.000 |
| SAS5 | 0.000 | 0.033 | 0.000 | 0.000 | 0.000 | 0.000 | 0.000 | 0.000 | 0.153 | 0.000 | 0.000 | 0.000 | 0.189 | 0.000 | 0.027 | 0.000 | 0.133 | 0.000 | 0.000 | 0.000 |
| SAS6 | 0.000 | 0.000 | 0.024 | 0.151 | 0.000 | 0.000 | 0.060 | 0.000 | 0.000 | 0.057 | 0.040 | 0.038 | 0.000 | 0.056 | 0.000 | 0.000 | 0.000 | 0.007 | 0.000 | 0.037 |
| SAS7 | 0.000 | 0.000 | 0.022 | 0.000 | 0.000 | 0.060 | 0.000 | 0.106 | 0.000 | 0.017 | 0.073 | 0.042 | 0.000 | 0.104 | 0.031 | 0.113 | 0.000 | 0.060 | 0.000 | 0.089 |
| SAS8 | 0.097 | 0.000 | 0.121 | 0.000 | 0.000 | 0.000 | 0.106 | 0.000 | 0.000 | 0.097 | 0.000 | 0.000 | 0.000 | 0.014 | 0.085 | 0.000 | 0.000 | 0.000 | 0.047 | 0.000 |
| SAS9 | 0.055 | 0.074 | 0.000 | 0.000 | 0.153 | 0.000 | 0.000 | 0.000 | 0.000 | 0.000 | 0.000 | 0.000 | 0.074 | 0.000 | 0.020 | 0.000 | 0.000 | 0.000 | 0.124 | 0.000 |
| SAS10 | 0.062 | 0.000 | 0.011 | 0.000 | 0.000 | 0.057 | 0.017 | 0.097 | 0.000 | 0.000 | 0.191 | 0.055 | 0.000 | 0.032 | 0.000 | 0.000 | 0.000 | 0.052 | 0.000 | 0.000 |
| SAS11 | 0.029 | 0.069 | 0.039 | 0.000 | 0.000 | 0.040 | 0.073 | 0.000 | 0.000 | 0.191 | 0.000 | 0.335 | 0.000 | 0.072 | 0.016 | 0.000 | 0.000 | 0.086 | 0.000 | 0.076 |
| SAS12 | 0.013 | 0.059 | 0.000 | 0.030 | 0.000 | 0.038 | 0.042 | 0.000 | 0.000 | 0.055 | 0.335 | 0.000 | 0.000 | 0.096 | 0.000 | 0.023 | 0.000 | 0.000 | 0.000 | 0.000 |
| SAS13 | 0.000 | 0.000 | 0.000 | 0.000 | 0.189 | 0.000 | 0.000 | 0.000 | 0.074 | 0.000 | 0.000 | 0.000 | 0.000 | 0.000 | 0.000 | 0.000 | 0.137 | 0.000 | 0.031 | 0.000 |
| SAS14 | 0.000 | 0.000 | 0.000 | 0.000 | 0.000 | 0.056 | 0.104 | 0.014 | 0.000 | 0.032 | 0.072 | 0.096 | 0.000 | 0.000 | 0.000 | 0.012 | 0.000 | 0.000 | 0.000 | 0.000 |
| SAS15 | 0.074 | 0.076 | 0.064 | 0.000 | 0.027 | 0.000 | 0.031 | 0.085 | 0.020 | 0.000 | 0.016 | 0.000 | 0.000 | 0.000 | 0.000 | 0.002 | 0.000 | 0.000 | 0.031 | 0.041 |
| SAS16 | 0.000 | 0.000 | 0.000 | 0.000 | 0.000 | 0.000 | 0.113 | 0.000 | 0.000 | 0.000 | 0.000 | 0.023 | 0.000 | 0.012 | 0.002 | 0.000 | 0.000 | 0.000 | 0.000 | 0.075 |
| SAS17 | 0.000 | 0.000 | 0.000 | 0.000 | 0.133 | 0.000 | 0.000 | 0.000 | 0.000 | 0.000 | 0.000 | 0.000 | 0.137 | 0.000 | 0.000 | 0.000 | 0.000 | 0.000 | 0.031 | 0.000 |
| SAS18 | 0.000 | 0.000 | 0.000 | 0.022 | 0.000 | 0.007 | 0.060 | 0.000 | 0.000 | 0.052 | 0.086 | 0.000 | 0.000 | 0.000 | 0.000 | 0.000 | 0.000 | 0.000 | 0.000 | 0.046 |
| SAS19 | 0.000 | 0.000 | 0.000 | 0.000 | 0.000 | 0.000 | 0.000 | 0.047 | 0.124 | 0.000 | 0.000 | 0.000 | 0.031 | 0.000 | 0.031 | 0.000 | 0.031 | 0.000 | 0.000 | 0.033 |
| SAS20 | 0.000 | 0.061 | 0.000 | 0.000 | 0.000 | 0.037 | 0.089 | 0.000 | 0.000 | 0.000 | 0.076 | 0.000 | 0.000 | 0.000 | 0.041 | 0.075 | 0.000 | 0.046 | 0.033 | 0.000 |

Table S3. Weighted adjacency matrix for depression symptoms among patients with chronic gastritis.

|  | SDS1 | SDS2 | SDS3 | SDS4 | SDS5 | SDS6 | SDS7 | SDS8 | SDS9 | SDS10 | SDS11 | SDS12 | SDS13 | SDS14 | SDS15 | SDS16 | SDS17 | SDS18 | SDS19 | SDS20 |
| --- | --- | --- | --- | --- | --- | --- | --- | --- | --- | --- | --- | --- | --- | --- | --- | --- | --- | --- | --- | --- |
| SDS1 | 0.000 | 0.000 | 0.171 | 0.114 | 0.068 | 0.000 | 0.021 | 0.000 | 0.026 | 0.193 | 0.005 | 0.017 | 0.248 | 0.022 | 0.140 | 0.000 | 0.000 | 0.015 | 0.000 | 0.000 |
| SDS2 | 0.000 | 0.000 | 0.000 | 0.000 | 0.033 | 0.175 | 0.000 | 0.000 | 0.000 | 0.000 | 0.032 | 0.041 | 0.000 | 0.019 | 0.000 | 0.000 | 0.000 | 0.010 | 0.000 | 0.000 |
| SDS3 | 0.171 | 0.000 | 0.000 | 0.000 | 0.033 | 0.000 | 0.000 | 0.000 | 0.000 | 0.000 | 0.000 | 0.000 | 0.083 | 0.000 | 0.044 | 0.000 | 0.000 | 0.000 | 0.039 | 0.057 |
| SDS4 | 0.114 | 0.000 | 0.000 | 0.000 | 0.000 | 0.000 | 0.000 | 0.083 | 0.000 | 0.000 | 0.000 | 0.000 | 0.076 | 0.000 | 0.047 | 0.000 | 0.000 | 0.000 | 0.047 | 0.000 |
| SDS5 | 0.068 | 0.033 | 0.033 | 0.000 | 0.000 | 0.129 | 0.034 | 0.000 | 0.000 | 0.065 | 0.070 | 0.000 | 0.025 | 0.000 | 0.000 | 0.000 | 0.000 | 0.025 | 0.009 | 0.056 |
| SDS6 | 0.000 | 0.175 | 0.000 | 0.000 | 0.129 | 0.000 | 0.000 | 0.000 | 0.000 | 0.000 | 0.032 | 0.146 | 0.050 | 0.049 | 0.010 | 0.005 | 0.027 | 0.096 | 0.000 | 0.007 |
| SDS7 | 0.021 | 0.000 | 0.000 | 0.000 | 0.034 | 0.000 | 0.000 | 0.000 | 0.000 | 0.048 | 0.000 | 0.000 | 0.149 | 0.000 | 0.094 | 0.000 | 0.000 | 0.000 | 0.073 | 0.000 |
| SDS8 | 0.000 | 0.000 | 0.000 | 0.083 | 0.000 | 0.000 | 0.000 | 0.000 | 0.000 | 0.089 | 0.000 | 0.000 | 0.000 | 0.000 | 0.000 | 0.000 | 0.000 | 0.000 | 0.000 | 0.000 |
| SDS9 | 0.026 | 0.000 | 0.000 | 0.000 | 0.000 | 0.000 | 0.000 | 0.000 | 0.000 | 0.086 | 0.000 | 0.000 | 0.059 | 0.000 | 0.048 | 0.000 | 0.000 | 0.000 | 0.034 | 0.000 |
| SDS10 | 0.193 | 0.000 | 0.000 | 0.000 | 0.065 | 0.000 | 0.048 | 0.089 | 0.086 | 0.000 | 0.000 | 0.000 | 0.050 | 0.000 | 0.063 | 0.000 | 0.000 | 0.050 | 0.035 | 0.000 |
| SDS11 | 0.005 | 0.032 | 0.000 | 0.000 | 0.070 | 0.032 | 0.000 | 0.000 | 0.000 | 0.000 | 0.000 | 0.192 | 0.000 | 0.107 | 0.062 | 0.129 | 0.092 | 0.011 | 0.000 | 0.000 |
| SDS12 | 0.017 | 0.041 | 0.000 | 0.000 | 0.000 | 0.146 | 0.000 | 0.000 | 0.000 | 0.000 | 0.192 | 0.000 | 0.000 | 0.095 | 0.000 | 0.119 | 0.000 | 0.056 | 0.017 | 0.118 |
| SDS13 | 0.248 | 0.000 | 0.083 | 0.076 | 0.025 | 0.050 | 0.149 | 0.000 | 0.059 | 0.050 | 0.000 | 0.000 | 0.000 | 0.031 | 0.163 | 0.000 | 0.000 | 0.015 | 0.084 | 0.000 |
| SDS14 | 0.022 | 0.019 | 0.000 | 0.000 | 0.000 | 0.049 | 0.000 | 0.000 | 0.000 | 0.000 | 0.107 | 0.095 | 0.031 | 0.000 | 0.000 | 0.108 | 0.235 | 0.089 | 0.000 | 0.187 |
| SDS15 | 0.140 | 0.000 | 0.044 | 0.047 | 0.000 | 0.010 | 0.094 | 0.000 | 0.048 | 0.063 | 0.062 | 0.000 | 0.163 | 0.000 | 0.000 | 0.000 | 0.000 | 0.005 | 0.083 | 0.000 |
| SDS16 | 0.000 | 0.000 | 0.000 | 0.000 | 0.000 | 0.005 | 0.000 | 0.000 | 0.000 | 0.000 | 0.129 | 0.119 | 0.000 | 0.108 | 0.000 | 0.000 | 0.068 | 0.022 | 0.000 | 0.155 |
| SDS17 | 0.000 | 0.000 | 0.000 | 0.000 | 0.000 | 0.027 | 0.000 | 0.000 | 0.000 | 0.000 | 0.092 | 0.000 | 0.000 | 0.235 | 0.000 | 0.068 | 0.000 | 0.243 | 0.000 | 0.102 |
| SDS18 | 0.015 | 0.010 | 0.000 | 0.000 | 0.025 | 0.096 | 0.000 | 0.000 | 0.000 | 0.050 | 0.011 | 0.056 | 0.015 | 0.089 | 0.005 | 0.022 | 0.243 | 0.000 | 0.000 | 0.279 |
| SDS19 | 0.000 | 0.000 | 0.039 | 0.047 | 0.009 | 0.000 | 0.073 | 0.000 | 0.034 | 0.035 | 0.000 | 0.017 | 0.084 | 0.000 | 0.083 | 0.000 | 0.000 | 0.000 | 0.000 | 0.000 |
| SDS20 | 0.000 | 0.000 | 0.057 | 0.000 | 0.056 | 0.007 | 0.000 | 0.000 | 0.000 | 0.000 | 0.000 | 0.118 | 0.000 | 0.187 | 0.000 | 0.155 | 0.102 | 0.279 | 0.000 | 0.000 |


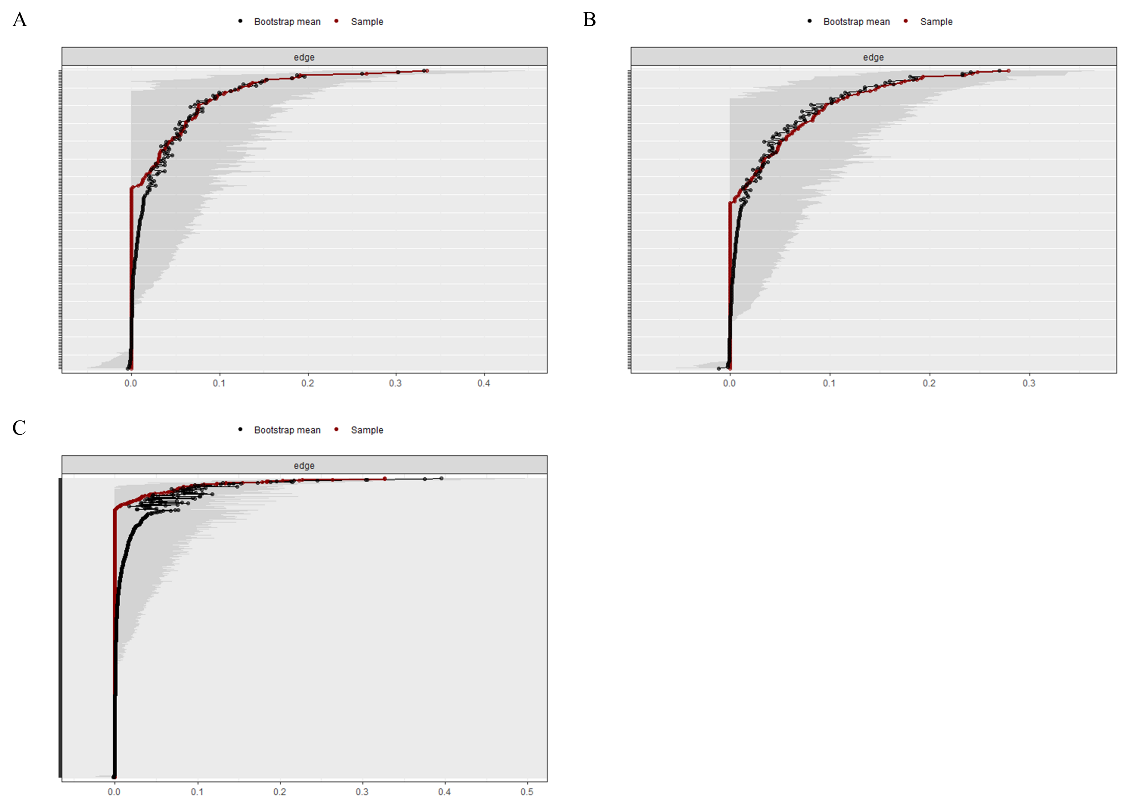


**Figure S1.** Nonparametric bootstrapped confidence intervals of estimated edges. The red line represents the estimated edge, while the shaded area indicates the 95% bootstrap confidence interval. A, anxiety for patients with chronic gastritis. B, depression for patients with chronic gastritis. C, anxiety-depression for chronic gastritis groups.


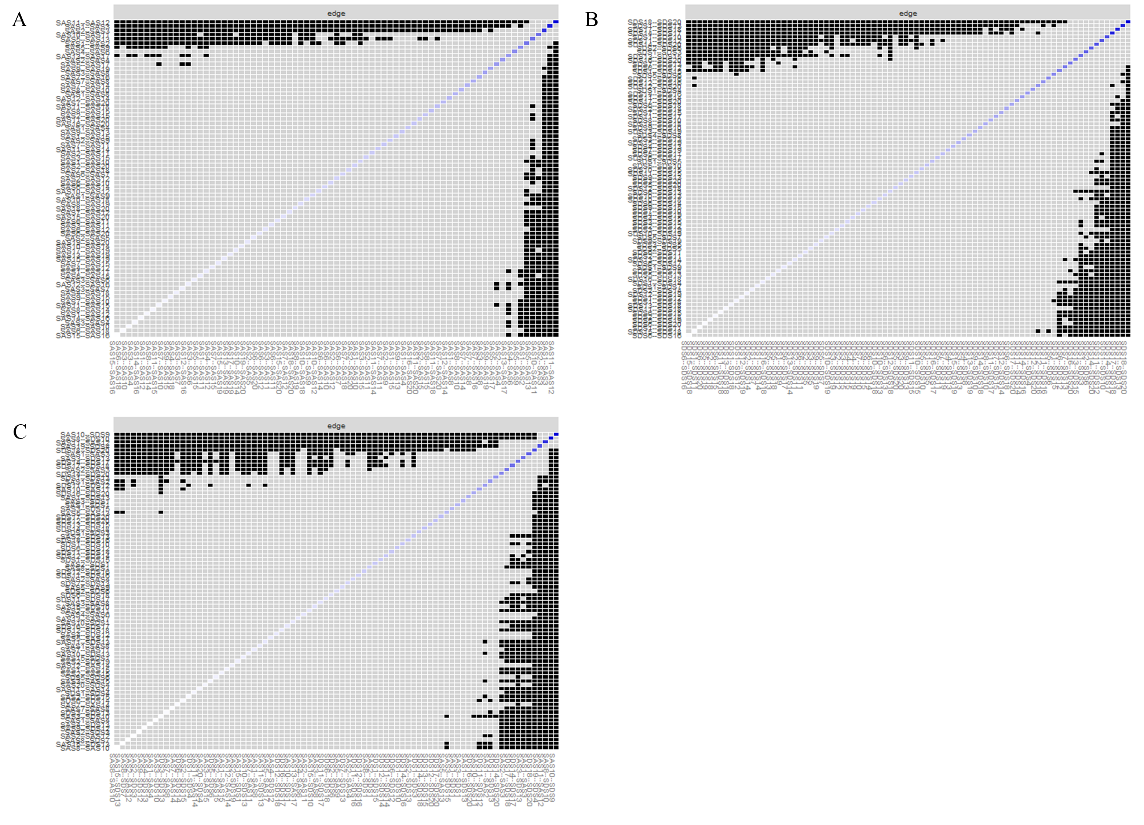


**Fig. S2.** Nonparametric bootstrapped difference test for edges. Grey boxes indicate no significant difference, whereas black boxes indicate a statistically significant difference (*p* < 0.05). Diagonal colour and saturation represent the magnitude and direction of each estimated edge. A, anxiety for patients with chronic gastritis. B, depression for patients with chronic gastritis. C, anxiety-depression for chronic gastritis groups.


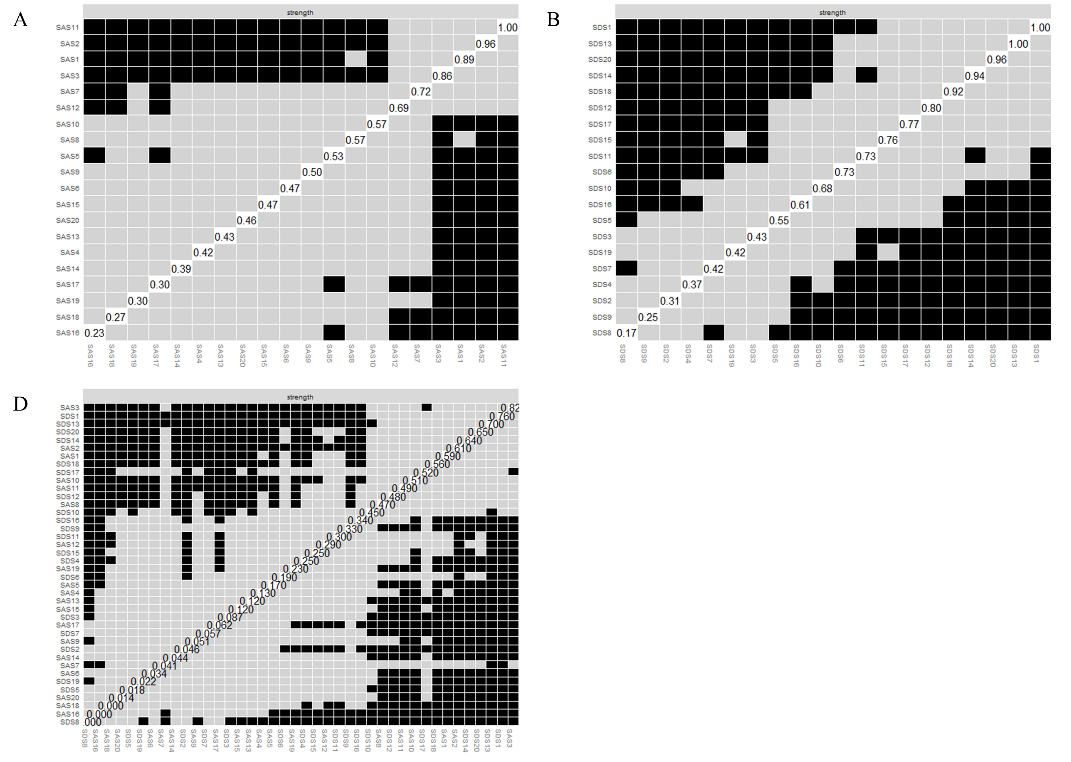


**Fig. S3.** The stability difference tests (α=0.05) for ‘node strength’. Grey boxes reflect no significant differences, and black boxes reflect significant differences. The number in the white boxes (i.e., the diagonal line) denotes the value of the node strength of a specific node. A, anxiety for patients with chronic gastritis. B, depression for patients with chronic gastritis. C, anxiety-depression for chronic gastritis groups.
